# The Antiepileptic Drug and Toxic Teratogen Valproic Acid Alters Microglia in an Environmental Mouse Model of Autism

**DOI:** 10.3390/toxics10070379

**Published:** 2022-07-09

**Authors:** Korawin Triyasakorn, Ubah Dominic Babah Ubah, Brandon Roan, Minsyusheen Conlin, Ken Aho, Prabha S. Awale

**Affiliations:** 1 Department of Biomedical and Pharmaceutical Sciences, College of Pharmacy, Idaho State University, 921 S 8th Avenue, Mail Stop 8288, Pocatello, ID 83209, USA; korawintriyasakor@isu.edu (K.T.); dominicbabahubah@isu.edu (U.D.B.U.); 2 Division of Health Sciences, Idaho State University, 921 S 8th Avenue, Mail Stop 8288, Pocatello, ID 83209, USA; brandonroan@isu.edu; 3 Department of Biological Sciences, Idaho State University, 921 S 8th Avenue, Mail Stop 8288, Pocatello, ID 83209, USA; sheenconlin@isu.edu (M.C.); kenaho1@gmail.com (K.A.)

**Keywords:** microglia, valproic acid, primary motor cortex

## Abstract

Autism spectrum disorder (ASD), a neurodevelopmental condition affecting approximately 1 in 44 children in North America, is thought to be a connectivity disorder. Valproic acid (VPA) is a multi-target drug widely used to treat epilepsy. It is also a toxic teratogen as well as a histone deacetylase inhibitor, and fetal exposure to VPA increases the risk of ASD. While the VPA model has been well-characterized for behavioral and neuronal deficits including hyperconnectivity, microglia, the principal immune cells of CNS that regulate dendrite and synapse formation during early brain development, have not been well-characterized and may provide potential hints regarding the etiology of this disorder. Therefore, in this study, we determined the effect of prenatal exposure to VPA on microglial numbers during early postnatal brain development. We found that prenatal exposure to VPA causes a significant reduction in the number of microglia in the primary motor cortex (PMC) during early postnatal brain development, particularly at postnatal day 6 (P6) and postnatal day 10 (P10) in male mice. The early microglial reduction in the VPA model coincides with active cortical synaptogenesis and is significant because it may potentially play a role in mediating impaired connectivity in ASD.

## 1. Introduction

Microglia are the principal resident immune cells of the CNS that colonize the brain during early prenatal development and comprise 5–15% of brain cells [1]. Recent advances in microglial and neuronal development have revealed they are implicated in all processes of neurogenesis and synaptogenesis including neuronal proliferation, migration, differentiation, as well as the formation and maturation of synaptic networks [2]. In early development, neurons make far more synaptic connections than are maintained in the adult brain. The large number of synapses that form in early development are eliminated by synaptic pruning, a developmental program that eliminates a large excess of synapses while also maintaining and strengthening a subset of them [3]. The precise colonization of microglia around areas undergoing active synaptogenesis during postnatal development strongly suggests that microglia and neuronal synapse formation may be influenced by each other. Indeed, several studies have shown that microglia interact with synapses, and this interaction is critical for synaptic maturation and synaptic connectivity. Microglia–synapse interaction occurs through several different pathways depending on the brain region. For example, the fractalkine receptor (CX3CR1) exclusively expressed by microglia is one mechanism involved in synaptic pruning. Genetically knocking out CX3CR1 in mouse hippocampus during postnatal development led to delayed synaptic pruning demonstrated by transient excess of dendritic spines that was also associated with decreased microglial numbers [4]. Another mechanism of microglia-mediated synaptic pruning in an activity and complement-dependent mechanism (CR3/CD11b-CD18/Mac-1) is found in the retinogeniculate system. Genetic (CR3 and C3 KO) and pharmacological perturbations specific to microglia resulted in sustained deficits in synaptic wiring in the postnatal dorso lateral geniculate nucleus (dLGN) of the thalamus [5]. These and other observations underscore the indispensable contribution and function of microglia in synaptic pruning and connectivity. Consequently, then, any chemicals, drugs or environmental agents that may potentially be toxic to microglia during the prenatal or postnatal period can adversely affect brain development, resulting in impaired synaptic connectivity and neurodevelopmental disorders such as autism.

Autism spectrum disorder (ASD), a neurodevelopmental disorder affecting nearly 1 in 54 children, is thought to result from aberrant brain connectivity [6] https://paperpile.com/c/hbo56Y/v8bG (accessed on 15 March 2022). Multiple studies suggest that brain connectivity in adults with ASD differs from young autistic children. In general, adults exhibit underconnectivity between brain areas engaged in cognitive tasks, while children exhibit functional hyperconnectivity at the whole brain and subsystems level, including sensory and association cortices [6,7]. Hyperconnectivity is also observed in animal models of autism including the valproic acid (VPA) model of autism. A model for idiopathic autism, the VPA model, has been well-characterized for behavioral deficits and neural deficits including neural hyperconnectivity in different brain regions [8,9,10]. The cause of this hyperconnectivity is largely unknown and may be due to the direct effect of VPA on neurons or as a consequence of the toxic effect of VPA on microglia or a combination of the two. The impact of prenatal VPA exposure on microglia in regions undergoing synaptogenesis in early postnatal development is also unknown and may provide some clues to the etiology of neural hyperconnectivity. Therefore, the current study seeks to first investigate the putative toxic effects of VPA on microglial changes in early postnatal brain development in a mouse model of autism. We hypothesize that prenatal exposure to VPA induces changes in the microglial population in this model. We demonstrate that prenatal exposure to VPA significantly reduces the microglial number in the primary motor cortex (PMC) at postnatal day 6 (P6) and P10 in male mice, a critical period coinciding with active synaptogenesis when microglia are essential for synaptic pruning. Together, these data suggest that environmental agents such as VPA induce changes in microglia, the crucial cellular machinery needed for all aspects of normal brain development. Such disruptions may profoundly affect synaptogenesis, synaptic pruning, synaptic maturation, and synaptic connectivity resulting in neurodevelopmental disorders, including autism.

## 2. Materials and Methods

### 2.1. Animals

Adult male and female BALB/c mice were paired for breeding. Female breeders were visually examined daily before 8:00 a.m. for the presence of a vaginal plug, which was recorded as day 0 of embryonic development (E0). Pregnant females were treated with either sterile saline or VPA (sodium valproate: Sigma, St. Louis, MO, USA) 600 mg/kg dissolved in sterile saline (filtered using a sterile Millex syringe filter Millipore Corporation, Bedford, MA, USA), injected subcutaneously (*s.c*) on E13 and administered at a volume of 10 mL/kg [11]. Day of birth was recorded as day 0 (P0). All animals were housed in the Northeast Ohio Medical University vivarium in temperature and humidity-controlled rooms under a 12 h/12 h light/dark cycle. Water and laboratory chow were available ad libitum. All NIH guidelines were strictly adhered to, and treatment of the mice was approved by the institutional animal care and use committee at Northeast Ohio Medical University. All efforts were made to minimize animal suffering, reduce the number of animals used, and to utilize alternatives to in vivo techniques, if available.

### 2.2. Tissue Collection

Male brains were collected at P6 and P10, *(n* = 6 brains/group; number of sections/brains used = 5). At the time of tissue collection, the mice were anesthetized (ketamine 100 mg/kg and xylazine 7.5 mg/kg) and perfused transcardially with cold 0.9% saline followed by 4% paraformaldehyde. The animals were decapitated, and the brains were removed and placed in cold 4% paraformaldehyde for 4 h, after which they were transferred into 20% sucrose for 24 h.

### 2.3. Immuno-Histochemical Detection of Iba1 Protein

Brains were sectioned (20 μm) coronally on a Leica cryostat at −20 °C, mounted directly onto Super frost ++ Micro slides (VWR), and allowed to dry before being stored at 4 °C. The ionized calcium-binding adaptor (Iba)-1 protein was used because its expression is constitutive in both activated (amoeboid) and quiescent microglia (ramified). The staining of all sections from all the groups was carried out under identical conditions. For staining, slides were removed from the freezer and allowed to thaw at RT for 10 min. The slides were washed with PBS and incubated for 1 h in PBS with 5% normal donkey serum (NDS) and 0.1% Triton X-100 to block and permeabilize the tissue. Following blocking, slides were incubated with primary antibody (rabbit anti-Iba1 at 1:500 dilution Wako Chemicals Richmond, VA, USA in 3% NDS and 0.1% Triton X-100) overnight at 4 °C. On day 2, the slides were washed and incubated with fluorescently labeled secondary antibody (Alexa Fluor 488 Donkey anti rabbit IgG H+L, at 1:500 dilution, Molecular Probes, USA in 1% NDS and 0.1% Triton X-100) for 2 h at RT in the dark. The slides were washed with PBS and incubated with the nuclear stain Red Dot 2 (far red nuclear dye: Biotium, CA, USA) for 20 min. The slides were washed and cover slipped with Fluoromount G (Southern Biotech, Birmingham, AL, USA) and dried overnight at RT.

### 2.4. Cell Counting

Iba1-labeled microglia were examined under a confocal microscope (Olympus IX 70) at 200× and at 600× magnification. The multi-laser feature on the confocal microscope and Flouview V 5.0 software were used to simultaneously acquire images of both microglia stained with Iba1 and the nuclei stained with Red Dot 2. This feature covers a wide spectrum of wavelengths ranging from 405 nm to 644 nm. An excitation wavelength of 488 nm with emission at 519 nm was used to acquire images of microglia fluorescently labeled with Alexa Fluor 488 nm. Red Dot 2 was excited at 634 nm with an emission at 695 nm. A systematic sampling of PMC area in each section was carried out. After the acquisition of images, the cells were counted using the cell counter feature of Image J (rsbweb.nih.gov/ij/ accessed on 24 April 2022) throughout each 20 µm section. Counting areas were set at of 800 × 600 µm, with a final magnification of 200×. To avoid counting cell fragments, cells were only counted as positive if the cell body was visible as yellow (merger of green and red) and the stain appeared uniform throughout the cell. For each animal, sections taken every 300 µm throughout the PMC region were analyzed. The number of representative sections analyzed for the PMC for the different groups was 5 sections/animal. The total number of Iba1-positive cells was obtained by an experimenter blind to experimental conditions, across all representative sections for the PMC visualized. The Atlas of the Developing Mouse Brain at E17.5, P0 and P10 (George Paxinos, Glenda Halliday, Charles Watson, Yuri Koutcherov, Hong Qin Wang) served as a reference guide to delineate the PMC of the sections used.

### 2.5. Statistical Analysis

The data obtained for the number of Iba1-positive microglia were averaged from 5 consecutive sections for each animal, and there were 6 animals (or 6 independent experiments from 6 different mothers) in each group, providing a sample size of 6. We tested for treatment effects using pooled variance *t*-test. Day 10 data required log transformation to meet linear model assumptions. Treatment effects were highly significant on both day 6 (*p* = 3.7 × 10^−5^) and particularly day 10 (*p* = 2.3 × 10^−9^). GraphPad Prism version 7.0 was used to draw the data figures and calculate statistical significance. The microglial number on the Y axis of the graphs was obtained by analyzing the number of microglia/area/µm^3^ (area = length × width × height) and converting the value obtained to mm^3^, which rendered the number of microglia/mm^3^. 

## 3. Results

### 3.1. VPA Treatment Reduces Microglial Number in the PMC of Male Mice at P6

**Figure 1:**
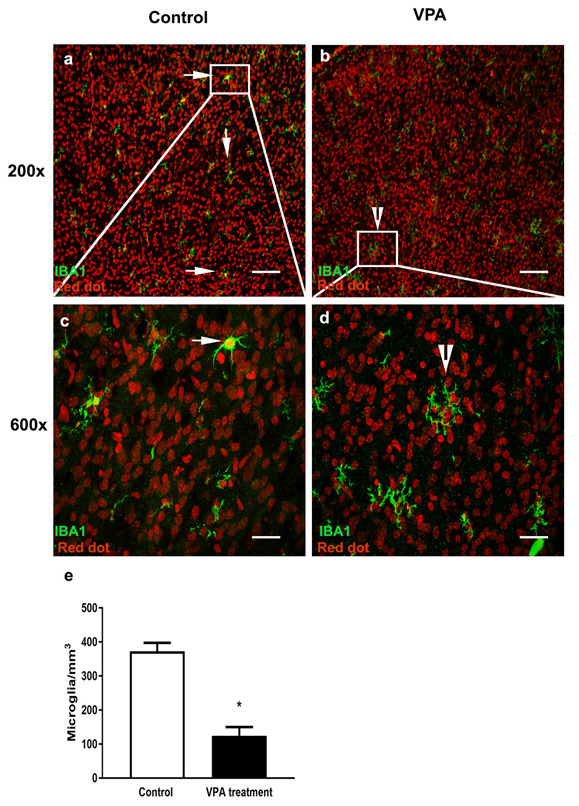
VPA-treated male mice show decreases in microglial number and fragmented morphology in primary motor cortex at P6. Confocal images showing labeled microglia in the PMC of saline control and age-matched brains of VPA mouse model of autism. (**a**,**b**) Microglia labeled with Iba1 (green) in control and VPA-treated mice at low magnification (200×). (**c**,**d**) Microglia labeled with Iba1 (green) at high magnification (600×). White square region (insert) in a and b is magnified in c and d. White arrows and arrowheads indicate differences in morphology of microglia in control and VPA model. Scale bar (**a**,**b**) 200 µm and in (**c**,**d**) 50 µm. (**e**) Analysis of number of microglia as summarized within the bar graphs (Mean ± SEM) reveals a statistically significant decrease in microglial number in VPA-treated mice (* *p* = 3.7 × 10^−5^).

The photomicrographs of Iba1-positive microglia (green) in the PMC (right side of the brain) from saline-treated and VPA-treated male mice at P6 are represented in Figure 1. Two times points were chosen for this study, namely P10 and P6. The P10 time point was selected based on the fact that in mice, P8–P16 constitutes the critical period of neuronal remodeling in the cerebral cortex [12]. We also wanted to determine the effect of VPA on microglia just before neuronal remodeling, and therefore, P6 was chosen. Because ASD disproportionately affects males compared to females with a 3:1 ratio, we focused our microglial studies on male animals. The primary motor cortex (PMC) was selected based on current literature that demonstrates complex arborizations of dendrites in PMC in the VPA rat model of autism [13]. In addition, children with autism also suffer from motor abnormalities as comorbid symptoms [14]. A summary of the data analyses of the number of microglia in the PMC is presented in the bar graphs shown in Figure 1e.

The number of microglia at P6 in VPA-treated mice (Mean ± SEM: 124 ± 25) was significantly lower than those found in saline-treated mice (Mean ± SEM: 372 ± 24). The microglia in the saline-treated mice appear to be healthy with a distinct cell body and nuclear staining (Figure 1a). At higher magnification (600×), the microglia appear to have characteristics of either amoeboid or intermediate morphology with short processes (Figure 1c). However, the majority of microglia in the VPA-treated mice upon close examination at higher magnification (600×) appear to be fragmented with no distinct cell body (Figure 1b,d).

### 3.2. VPA Treatment Depletes Microglia in PMC of Male Mice at P10

**Figure 2:**
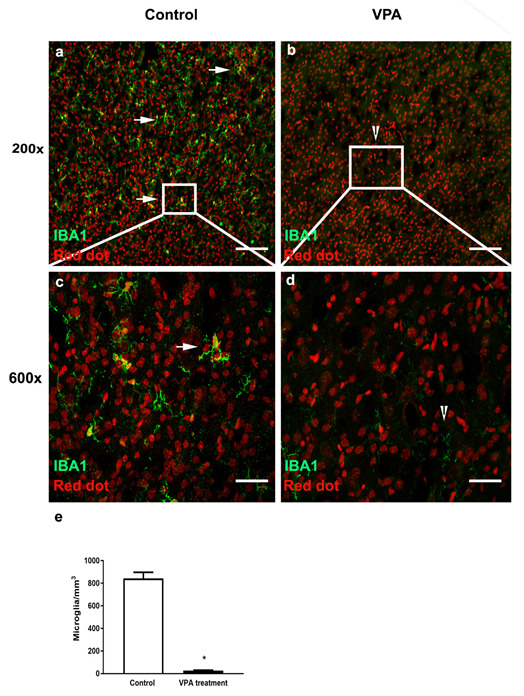
VPA-treated male mice show significant decrease in microglial number and fragmented morphology in primary motor cortex at P10. Confocal images showing labeled microglia in cortex of saline control and age matched brains in VPA mouse model of autism. (**a**,**b**) Microglia labeled with Iba1 (green) in control mice and VPA-treated mice at low magnification (200×). (**c**,**d**) Microglia labeled with Iba1 (green) at high magnification (600×). White square region (insert) in a and b is magnified in c and d. White arrows and arrowheads indicate differences in morphology of microglia in control and VPA model. Scale bar (**a**,**b**) 200 µm and in (**c**,**d**) 50 µm. (**e**) Analysis of number of microglia as summarized within the bar graphs (Mean ± SEM) reveals a statistically significant decrease in microglial number in VPA-treated mice (* *p* = 2.3 × 10^−9^).

The photomicrographs of Iba1 positive microglia (green) in the PMC from saline-treated and VPA-treated male mice at P10 are represented in Figure 2. A summary of the data analyses of the number of microglia in the PMC is presented in the bar graphs of Figure 2e.

The number of microglia at P10 in VPA-treated mice (Mean ± SEM: 26 ± 3) was significantly lower than saline-treated mice (Mean ± SD: 824 ± 54). The microglia in the saline-treated mice appear to be healthy with a distinct cell body and nuclear staining (Figure 2a). At higher magnification (600×), the microglia appear to have characteristics of either an intermediate morphology with short processes or ramified morphology with thin processes and a distinct cell body (Figure 2c). In contrast, the microglia in the VPA-treated mice appear to have been completely depleted by the treatment (Figure 2b). Even upon close examination at higher magnification (600×), very few fragments are observed with no distinct cell body (Figure 2d).

## 4. Discussion

To our knowledge, this study marks the first attempt to investigate the effect of embryonic exposure to VPA on microglial number particularly during early postnatal development in a male VPA mouse model of autism. We demonstrate that embryonic exposure to VPA has distinct effects on the microglial number during early postnatal period of brain development. Specifically, we demonstrate reduced microglial number and fragmented morphology at P6 and significantly lower microglial numbers at P10, particularly in male offspring.

The current study analyzed the early postnatal period of brain development to more closely match the period of early symptoms seen in humans with ASD. Clinically, autism is associated with impairments in social behavior, verbal and nonverbal communication and stereotypical repetitive behaviors and narrowed interest [15,16]. One of the hallmarks of autism is hyperconnectivity, found to be higher in children than in adults, inferring neurodevelopmental origin, the cause of which is elusive, but it is hypothesized that a lack of pruning of synapses may be responsible for impairments in connectivity [6,7]. Microglia are key players in pruning extranumerary synapses in development [3]. VPA-exposed animals exhibit impaired communication, reduced exploratory behavior, and anxiety-like and repetitive behaviors, a phenotype that models behavioral impairments in children with autism [17]. Multiple reports in the literature have demonstrated either an increase, decrease or no change in microglial density in different neuroanatomical regions, at later time points in postnatal development in both male and female offspring exposed to VPA prenatally. Of particular interest is the observation that no changes in microglial density were observed in the Dentate Gyrus (DG) (molecular layer, granular cell layer, Hilus), CA1 region of the hippocampus (stratum oriens, pyramidal cell layer, stratum radiatum) and cerebellum (molecular layer, granular cell layer) at P7 in a female VPA model of autism [18]. This is in contrast to our data that show fragmented microglia at P6 and near complete depletion of microglia during peak period of synaptogenesis and synaptic maturation at P10 in the PMC in a male VPA model, highlighting age-specific differences, region-specific differences and sexual differences to drug response. It is also important to note that the mouse strains used in these studies are different from what we used. It is possible that microglia in BALBc mice may be particularly susceptible to the deleterious effect of VPA.

The reduction in microglia number close to the time of insult we observe in our experiments is also in contrast to other studies in young adult animals at a time point after remodeling occurs in the brain. For example, increased microglial density has been reported in the molecular layer of the dentate gyrus in female BALBc mice at 5 months [19]. This is in contrast to studies by Kazlauskas et al., who showed no change in microglial density in the same molecular layer of the dentate gyrus; however, this was carried out in early neonatal (PD-7) development and in a different strain Cr1Fcen:CF1 lineage showing age-specific and strain specific effects of the drug [18].

In yet another interesting report, Gassowska-Dobrowolska et al. studied changes induced by VPA on protein expression of IBA-1 a microglial marker and mRNA levels of pro and anti-inflammatory markers in the cerebral cortex and hippocampus at PD58 in rats. Prenatal exposure to VPA leads to a significant increase in protein levels of IBA1 in the cerebral cortex, which may reflect a higher microglial number. Additionally, the mRNA levels of proinflammatory cytokines (IL-1β, IL-6. TNF-α), and anti-inflammatory neuroprotective phenotypes (Arg1, Chi3L1, Mrc1, CD86, Fcgr1a, TGFβ1, and Sphk1) were increased. On the other hand, the same VPA treatment resulted in no change in the above parameters, except for the anti-inflammatory and neuroprotective phenotype that showed an increase in the hippocampus, indicating ongoing immune system impairments, which may be more robust in the cortex compared to the hippocampus [17]. An anti-inflammatory phenotype in the hippocampus may reflect the possible resolution of immune activation that may have started earlier than in the cerebral cortex. These observations also correlate well with observations seen in the clinical symptoms of autism. For example, in high-functioning males with ASD, there is an elevated plasma level of several cytokines IL-1β, IL-1RA, IL-5, IL-8, IL-12, (p70), IL-13, and IL-17 [20]. In addition to increases in the proinflammatory cytokine Il-1β, another proinflammatory cytokine TNF-α is also found to be elevated in the cerebrospinal fluid of ASD [21]. There are frequent reports of an increase in IL-6 both centrally and peripherally in ASD patients [22,23,24,25]. Interestingly, researchers have noticed an association between peripheral cytokine levels and the severity of behavioral impairments. For example, elevated IL-1β and IL-6 are associated with increased stereotypical behavior [25], and the dysregulation of IL-1β is associated with impairments in memory and learning [26]. Reduced levels of the regulatory cytokine TGFβ are associated with reduced adaptive behavior and worsening behavioral symptoms [27]. Elevated levels of IL-8 and IL-12p40 are also associated with greater impairment of aberrant behavior including lethargy and stereotypy. Now, as the expression of IL-8 decreases, cognitive and adaptive ability improves [25]. All these studies indicate that ASD patients are in a state of continuous immune dysregulation, which impacts behavior, although many of these studies are carried out in either adult animals or humans. The immune response of microglia in different brain regions to VPA in early development in both inbred and outbred strains of mice remains to be fully elucidated and may be useful in determining the pathology of ASD.

It is unclear how prenatal exposure to VPA reduces microglia during early postnatal development, which remains to be fully elucidated. One possibility is that VPA induces microglial apoptosis, and it is likely the fragments at P6 are apoptotic bodies. Interestingly, at P10, we observe a near complete absence of microglia including fragments. There is some evidence from cell culture studies where VPA selectively killed cultured murine BV-2 microglia by a caspase 3-mediated mechanism, sparing the neurons and astrocytes, suggesting that this might be one possible mechanism [28]. A second possibility is that VPA induces microglial necrosis. Alternatively, a third possibility is that VPA reduces microglial cell proliferation. Regardless of the mechanism, VPA’s deleterious and toxic impact on microglial number particularly during the critical window of synaptogenesis raises the possibility that this might potentially have an impact on neuronal synaptogenesis, so the further exploration of cause and effect would be of interest. Contrary to adulthood where either an increase, decrease or no changes in microglial density are observed, our results demonstrate microglial reduction may be an early effect of VPA. It is noteworthy that impaired microglial function in mice has been shown to induce behavioral deficits related to clinical symptoms of ASD, obsessive compulsive disorder and schizophrenia [29].

ASD is thought to result from aberrant hyperconnectivity. There is also evidence for general hyperconnectivity in the somatosensory cortex, medial prefrontal cortex (mPFC), amygdala and auditory cortex in the rodent VPA model of ASD [8,9,10]. Although strains used in these studies are different, a clear picture of hyperconnectivity is emerging, the underlying cause of which is not clear, but dysfunctional/depleted microglia may be a key player in hyperconnectivity in ASD. Future experiments should aim to test the hypothesis that microglial dysfunction alters synaptic connectivity in the VPA model of autism. One limitation of our study is that we performed these experiments in male mice only. In the future, it is equally important to study the effect of VPA on female mice as well.

In summary, the present experiments are the first to show that, in mammals, VPA reduces the microglial number particularly during critical periods of synaptogenesis in PMC. The data reported in this study are important and relevant to pathological states associated with autism. Clearly, this is an interesting area that merits further investigation.

## Data Availability

Not applicable.
